# Subcutaneous Furosemide for Ambulatory Decongestion in Heart Failure: Outcomes From a High‐Risk Real‐World Cohort

**DOI:** 10.1002/clc.70514

**Published:** 2026-11-03

**Authors:** Omar Jafar, Ann Harper, Angela Toepp, Amin Yehya

**Affiliations:** ^1^ Internal Medicine Macon & Joan Brock Virginia Health Science at Old Dominion University Norfolk Virginia USA; ^2^ Sentara Enterprise Analytics Vigrinia Beach Virginia USA; ^3^ Cardiology Macon & Joan Brock Virginia Health Science at Old Dominion University Norfolk Virginia USA

## Abstract

**Background:**

Volume overload remains a primary driver of heart failure hospitalization (HFH). Diuretic resistance limits the efficacy of oral loop diuretics during decompensation, often necessitating intravenous therapy. Subcutaneous (SC) furosemide (Furoscix, 80 mg/10 mL) represents a novel outpatient alternative for patients who remain symptomatic despite oral diuretic therapy.

**Objectives:**

To evaluate the clinical outcomes of SC furosemide in ambulatory heart failure (HF) patients inadequately managed with oral loop diuretics.

**Methods:**

We performed a single‐center retrospective cohort study of HF patients prescribed SC furosemide as an adjunct to oral loop diuretics between May 2023 and 2025. The primary outcome was HFH within 30 days of initiation. Secondary outcomes included a composite of HFH or urgent care or emergency department visits requiring intravenous diuresis, change in body weight, and New York Heart Association (NYHA) functional class.

**Results:**

Fifty patients met the inclusion criteria; one was excluded for incomplete follow‐up. SC furosemide was prescribed as two to three doses per week. Within 30 days, 10 patients (20.4%) experienced HFH; six had been hospitalized in the preceding 30 days, suggesting a high‐risk baseline cohort. The composite endpoint occurred in 16 patients (32.7%). Among patients who avoided hospitalization, median NYHA class improved from III to II, and mean body weight decreased by 2.6 kg (5.7 lbs). Adverse events were infrequent; no deaths occurred within the 30‐day follow‐up period.

**Conclusion:**

In ambulatory HF patients with persistent congestion despite oral diuretic therapy, SC furosemide was well tolerated and associated with improvements in functional status and volume burden.

## Graphical abstract

In 49 high‐risk ambulatory heart failure patients with persistent congestion despite oral diuretics, subcutaneous furosemide was well tolerated, with a 20.4% 30‐day HF hospitalization rate. Among non‐hospitalized patients, the median NYHA class improved from III to II, and the weight decreased by 2.6 kg.

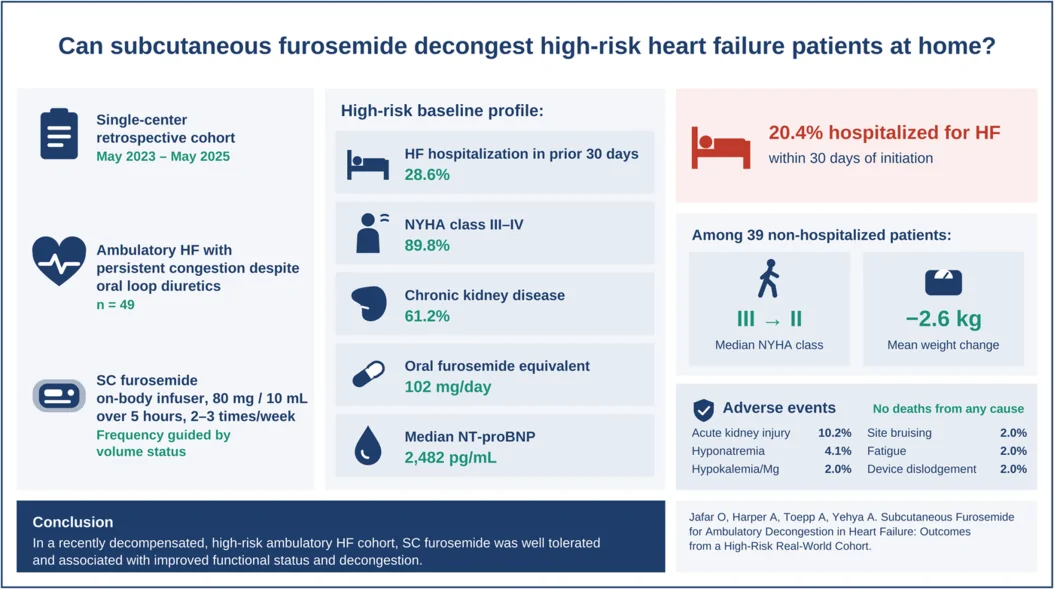

## 1 Introduction

Heart failure (HF) affects greater than 6.7 million adults in the United States (US) and carries substantial morbidity, mortality, and healthcare resource utilization [1]. It is among the leading causes for emergency department (ED) visits and hospital admissions in adults, accounting for 1 272 855 primary hospitalizations in the United States in 2023 [2], and more than 80% of patients presenting to the ED with acute HF are admitted [3].

Guideline‐directed medical therapy (GDMT) has substantially reduced HF hospitalization and mortality in clinical trials [4]. Nevertheless, episodes of congestion necessitating intravenous (IV) diuretic therapy remain a leading driver of ED visits and unplanned admissions [3, 5, 6]. These episodes do not only strain healthcare infrastructure but also adversely affect patients' quality of life and disease trajectory.

The 2022 American Heart Association/American College of Cardiology/Heart Failure Society of America (AHA/ACC/HFSA) Heart Failure Guidelines recommend loop diuretics as first‐line therapy for volume overload in patients with HF [7]. During decompensation, diuretic resistance can limit the efficacy of oral loop diuretics, often necessitating IV administration. A pH‐neutral formulation of furosemide (80 mg/10 mL; Furoscix), delivered subcutaneously (SC) by an on‐body infuser, has emerged as an alternative to IV therapy, that allows decongestion in an ambulatory setting [8].

We therefore conducted a single‐center retrospective study to evaluate the feasibility, safety, and short‐term clinical outcomes of SC furosemide in patients with HF who remained symptomatic and volume‐overloaded despite oral diuretic therapy.

## 2 Methods

### 2.1 Study Design and Population

We conducted a single‐center retrospective cohort study of patients with HF who were prescribed SC furosemide (Furoscix, 80 mg/10 mL) as an adjunct to oral loop diuretic therapy between May 2023 and 2025. Patients were eligible if they were 18−85 years of age, had a documented diagnosis of HF, were receiving daily oral loop diuretic therapy, and had received at least one course of SC furosemide during the study period. SC furosemide was prescribed at the discretion of the treating HF clinician for patients with persistent volume overload despite uptitration of oral loop diuretic therapy, with the aim of preventing heart failure hospitalization (HFH) or urgent care/ED visits. The index date was defined as the date of the first SC furosemide prescription. The study was approved by the Institutional Review Board, which waived the requirement for informed consent given the retrospective design.

### 2.2 Data Collection

Baseline characteristics extracted from the electronic health record and included demographic data, left ventricular ejection fraction (LVEF) from the most recent echocardiogram closest to SC furosemide initiation, New York Heart Association (NYHA) functional class at the time of prescription and at 30 days, current GDMT, oral loop diuretic regimen, relevant comorbidities, N‐terminal pro–B‐type natriuretic peptide (NT‐proBNP) at the time of prescription, and body weight at baseline and 30 days.

Oral loop diuretic doses were recorded and standardized to oral furosemide equivalents using established conversion factors (furosemide 40 mg = torsemide 20 mg = bumetanide 1 mg) [7]. Oral loop diuretic doses reflect the regimen prescribed at the SC furosemide initiation visit, including any concurrent dose adjustment. All‐cause mortality and HF events, including those at outside institutions, were ascertained through review of the electronic health record and linked external records via Epic Care Everywhere.

### 2.3 Outcomes

The primary outcome was HFH within 30 days of SC furosemide initiation. Secondary outcomes included a composite of HFH or an ED visit or urgent care visit requiring IV diuresis, all‐cause mortality within 30 days, change in body weight, and change in NYHA functional class from baseline to 30 days, and other adverse events. Adverse events included (infusion‐site reactions, device malfunction, acute kidney injury [AKI], hypokalemia [K < 3.5 mEq/L], hyponatremia [Na < 135 mEq/L], and symptomatic hypotension). AKI was defined using modified Kidney Disease: Improving Global Outcomes (KDIGO) serum creatinine (Cr) criteria as an increase of ≥ 0.3 mg/dL or ≥ 1.5 times baseline [9]. Because of the outpatient setting, the 48 h and 7‐day windows could not be strictly applied. Instead, AKI was assessed using the first Cr measured after the index date. Baseline laboratory values were the most recent measurements before or on the index date. Follow‐up values were the first measurements after the index date, if obtained.

### 2.4 Statistical Analysis

Continuous variables are reported as mean ± standard deviation or median (interquartile range) according to distribution. Categorical variables were reported as counts and percentages. The proportion of patients with ≥ 1 HFH in the 30 days before and after SC furosemide initiation was compared using the exact McNemar test.

## 3 Results

### 3.1 Patient Characteristics

Fifty patients met the inclusion criteria, one patient was excluded due to incomplete follow‐up data, leaving 49 patients for analysis (Figure 1). Baseline characteristics are summarized in Table 1. The mean age was 62.4 ± 15.5 years, and 55% were male. The cohort included 46.9% Black or African American patients and 46.9% White patients. Mean LVEF was 39.7 ± 19.1%. By LVEF, 27 patients (55.1%) had HFrEF, 6 (12.2%) had HFmrEF, and 16 (32.7%) had HFpEF. Comorbidities included: chronic kidney disease (CKD), defined as an estimated glomerular filtration rate of < 60 mL/min/1.73 m^2^, was present in 61.2%, chronic lung disease in 61.2%, and Type 2 diabetes mellitus in 61.2%. All patients were receiving oral diuretics at baseline, with a mean daily furosemide equivalent dose of 102 ± 65 mg. Oral loop diuretic doses were concurrently increased at the initiation visit in 15 patients (30.6%), including 11 of 39 (28.2%) non‐hospitalized and 4 of 10 (40.0%) hospitalized patients. GDMT was concurrently modified at the initiation visit in 6 patients (12.2%), with additions of: SGLT2 inhibitor (*n* = 3), mineralocorticoid receptor antagonist (*n* = 2), and sacubitril/valsartan (*n* = 1). At SC furosemide initiation, 44 patients (89.8%) were in NYHA class III or IV (class III, *n* = 40; class IV, *n* = 4).

**Figure 1:**
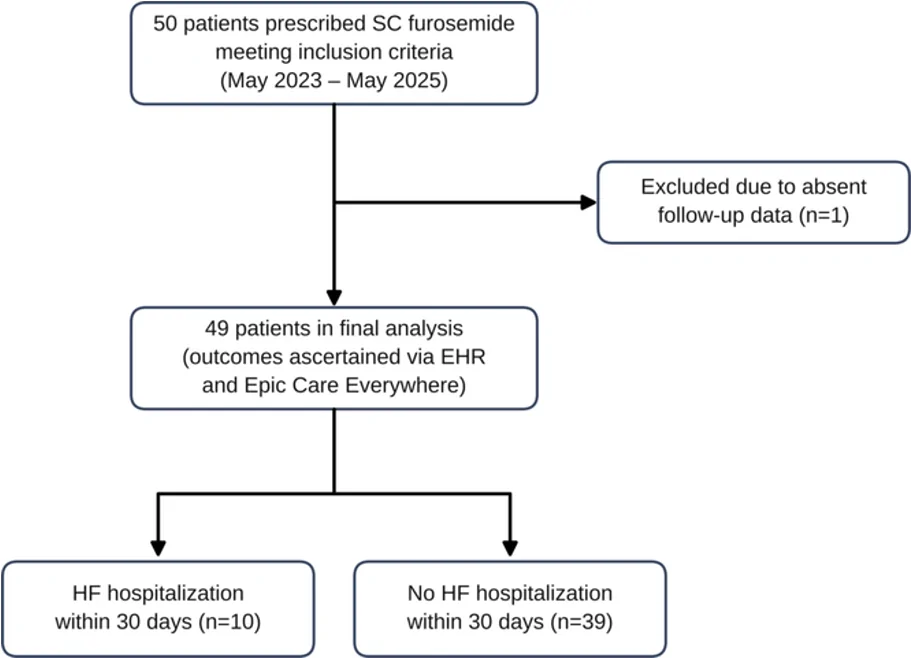
Study flow diagram. Fifty patients prescribed subcutaneous (SC) furosemide met the inclusion criteria; one was excluded due to absent follow‐up data, leaving 49 patients for analysis. Outcomes were ascertained through the electronic health record (EHR) and linked external records via Epic Care Everywhere. HF, heart failure.

**Table 1:** Baseline characteristics by hospitalization status within 30 days.

Characteristic	Non‐hospitalized (*n* = 39)	Hospitalized (*n* = 10)
Demographics		
Age, years	62.36 ± 16.65	62.7 ± 10.31
Male sex	21 (53.8%)	6 (60%)
Race/ethnicity		
Black or African American	17 (43.6%)	6 (60%)
White	19 (48.7%)	4 (40%)
Other	3 (7.7%)	0
Cardiac parameters		
LVEF, %	42.54 ± 18.41	28.40 ± 18.56
Baseline NT proBNP, pg/mL^a^	1654 (850−4328)	11 748 (2289−15 817)
Medical history		
Chronic kidney disease*	22 (56.4%)	8 (80%)
Chronic lung disease	23 (59.0%)	7 (70%)
Type 2 diabetes	23 (59.0%)	7 (70%)
Diuretic therapy		
Furosemide	14 (35.9%)	1 (10%)
Torsemide	7 (17.9%)	4 (40%)
Bumetanide	18 (46.2%)	5 (50%)
Daily furosemide equivalent, mg^b^	98.46 ± 66.86	116 ± 57.96
Guideline‐directed medical therapy^c^		
HFpEF (LVEF ≥ 50%)	15	1
SGLT2 inhibitors	7 (46.7%)	0
MRA	10 (66.7%)	0
HFrEF/HFmrEF (LVEF < 50%)	24	9
Beta blockers	22 (91.7%)	8 (88.9%)
ACE/ARB/ARNi	14 (58.3%)	6 (66.7%)
MRA	17 (70.8%)	5 (55.6%)
SGLT2 inhibitors	16 (66.7%)	6 (66.7%)

*Note:* Values are mean ± SD, median (IQR), or *n* (%). Defined as an estimated glomerular filtration rate (eGFR) < 60 mL/min/1.73 m^2^

Abbreviations: ACEi, angiotensin‐converting enzyme inhibitors; ARB, angiotensin receptor blocker; ARNI, angiotensin receptor‐neprilysin inhibitor; HFmrEF, heart failure with mildly reduced ejection fraction; HFpEF, heart failure with preserved ejection fraction; HFrEF, heart failure with reduced ejection fraction; LVEF, left ventricular ejection fraction; MRA, mineralocorticoid antagonist; NT‐proBNP, N‐terminal pro‐B‐type natriuretic peptide; SGLT2, sodium‐glucose cotransporter 2.

^a^ NT‐proBNP available in 30/49 patients (5 hospitalized, 25 non‐hospitalized).

^b^ Loop diuretic doses were converted to furosemide equivalents with 1 mg bumetanide = 20 mg torsemide = 40 mg furosemide.

^c^ GDMT is reported by phenotype‐appropriate indication, with percentages calculated within each phenotype group; LVEF < 50% includes HFmrEF (*n* = 6).

NT proBNP values at the time of prescription were available in 30 of 49 patients (61.2%), with an overall median of 2482 pg/mL (IQR 1003−4737), including five patients subsequently hospitalized and 25 who were not. Among hospitalized patients, median NT proBNP was 11 748 pg/mL (IQR 2289−15 817), compared with 1654 pg/mL (IQR 850−4328) in the non‐hospitalized group, reflecting markedly increased disease burden and increased baseline risk in those who subsequently required hospitalization (Figure 2).

**Figure 2:**
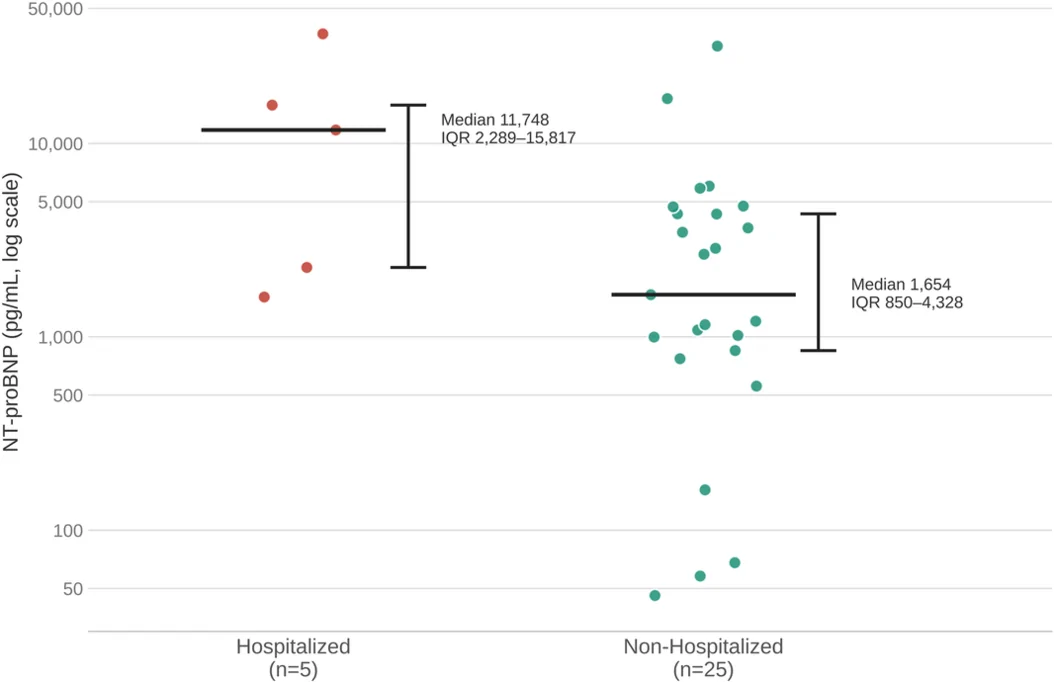
Baseline NT‐proBNP by 30‐day heart failure hospitalization status. NT‐proBNP levels were available in 30/49 patients (5 hospitalized, 25 non‐hospitalized). Each point represents one patient; horizontal lines indicate medians, and error bars indicate interquartile ranges. The *y*‐axis is on a logarithmic scale.

### 3.2 SC Furosemide Administration

SC furosemide (80 mg/10 mL) was administered via a single‐use, on‐body infuser delivering 30 mg over the first hour, followed by 12.5 mg/h for 4 h. It was prescribed two to three times weekly, and patients were instructed to withhold their morning oral loop diuretic dose on administration days to reduce the risk of excessive diuresis; patients on twice‐daily regimens continued the evening dose.

### 3.3 Primary Outcome

Within 30 days of SC furosemide initiation, 10 patients (20.4%) experienced HF hospitalization. Notably, six of these 10 patients had been hospitalized for HF in the 30 days preceding SC furosemide initiation, underscoring the high‐risk clinical profile of this cohort. In the 30 days preceding SC furosemide initiation, 14 patients (28.6%) were hospitalized for HF, compared with 10 (20.4%) in the 30 days following initiation (exact McNemar *p* = 0.39).

### 3.4 Secondary Outcomes

The composite outcome of HFH or an ED visit or urgent care visit requiring IV diuresis occurred in 16 patients (32.7%), comprising 18 events: 10 HFH, seven urgent care visits, and one ED visit. One urgent care visit occurred in a patient subsequently hospitalized, and the ED visit occurred in a patient with a prior urgent care visit; each patient was counted once.

Among the 39 patients without HFH, median NYHA class improved from III to II, and mean weight decreased by 2.6 kg (5.7 lbs) (Figure 3).

**Figure 3:**
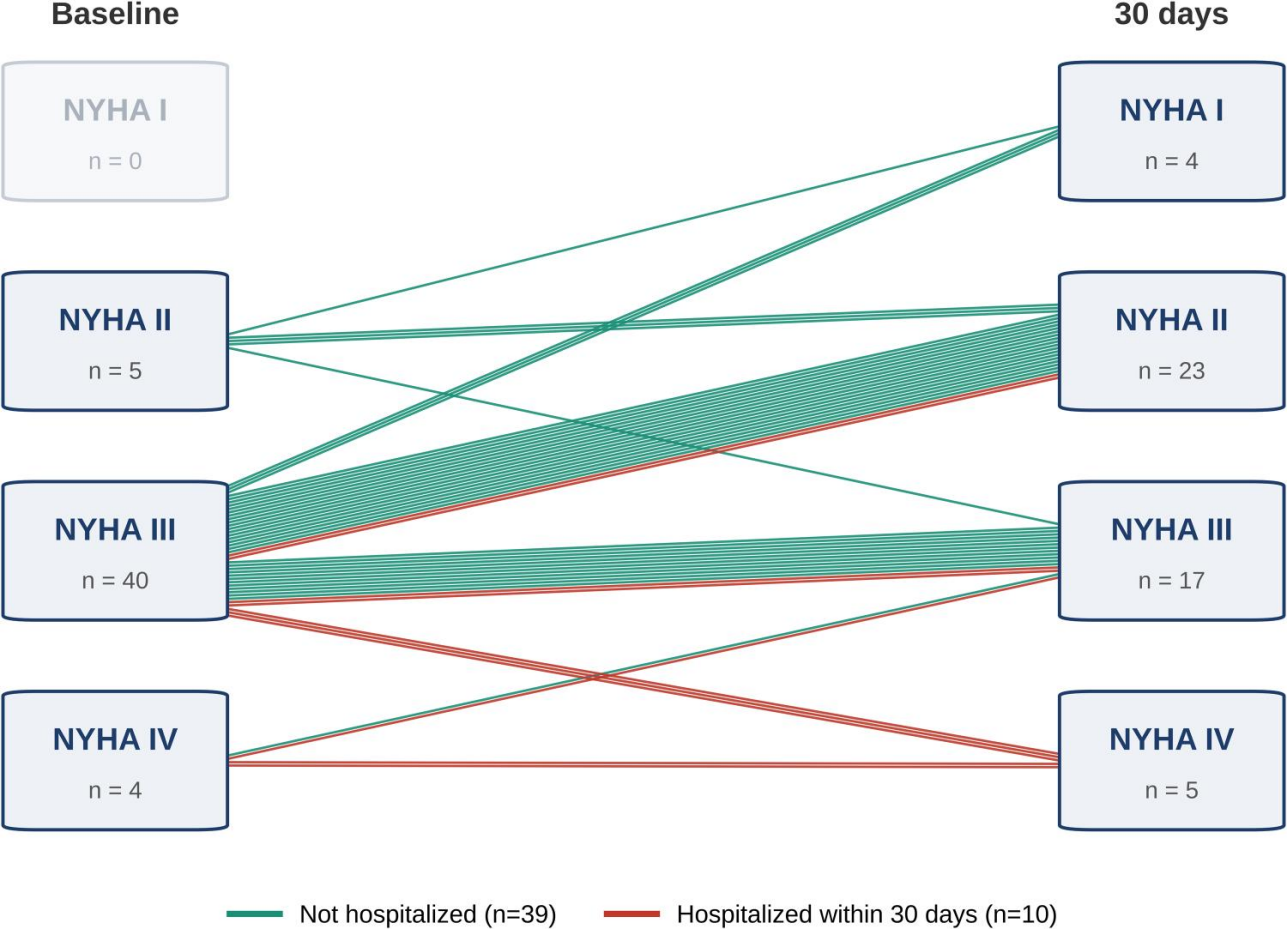
Individual changes in NYHA functional class at baseline and 30 days. Each line represents one patient (*n* = 49); green indicates patients not hospitalized during follow‐up (*n* = 39), and red indicates patients hospitalized for heart failure within 30 days (*n* = 10). NYHA class at 30 days in hospitalized patients reflects post‐hospitalization status. Numbers indicate patients in each class at each time point.

### 3.5 Adverse Events

SC furosemide was generally well tolerated. Adverse events occurred in nine patients (18.4%). Among the nine patients, AKI occurred in 5 (10.2%), hyponatremia in 2 (4.1%), hypokalemia with hypomagnesemia in 1 (2.0%), bruising in 1 (2.0%), fatigue in 1 (2.0%), and device dislodgement in 1 (2.0%). No patients died during follow‐up.

## 4 Discussion

In this single‐center retrospective cohort study, we report the real‐world experience of 49 high‐risk patients with HF who remained symptomatic and volume overloaded despite oral diuretic therapy and subsequently treated with SC furosemide (Furoscix, 80 mg/10 mL) in the outpatient setting. Our findings suggest that SC furosemide is well tolerated and associated with improvements in functional status and decongestion among patients who did not require hospitalization. These results are particularly noteworthy given the markedly high‐risk profile of the cohort and add to a growing body of evidence supporting SC furosemide as a viable outpatient decongestion strategy in patients who remained symptomatic despite escalation of oral diuretics.

This cohort represents a high‐risk population. All patients remained symptomatic despite oral diuretic therapy and would otherwise typically have been referred to the ED or urgent care for IV diuretics. The mean daily loop diuretic dose was 102 ± 65 mg daily in furosemide equivalents; nearly 90% were in NYHA class III or IV at initiation, the median NT‐proBNP was 2482 pg/mL, and 14 of 49 patients (28.6%) had been hospitalized for HF in the preceding 30 days. The 30‐day HFH rate of 20.4% should be interpreted in this context. Hospitalizations were concentrated among patients with a recent decompensation: six of the 10 patients hospitalized during follow‐up had been admitted for HF in the 30 days before SC furosemide initiation.

Comorbidity burden was also higher in hospitalized patients: 8 (80%) had CKD, 7 (70%) chronic lung disease, and 7 (70%) type 2 diabetes. Median NT‐proBNP at initiation was substantially higher in patients subsequently hospitalized than those who were not (11 748 vs. 1654 pg/mL), consistent with the established association between elevated NT‐proBNP and adverse HF outcomes [10]. This association may be amplified in patients with CKD, in whom NT‐proBNP elevations have been linked to greater risk of HFH and cardiovascular death [11], a relevant consideration given the prevalence of CKD in our cohort. However, NT proBNP was available only in 30 patients, including five who were hospitalized; the groups were not formally compared. These findings are therefore hypothesis‐generating, but they suggest that baseline NT‐proBNP could help identify patients unlikely to respond to outpatient SC furosemide who may benefit from earlier escalation of care.

When assessed against phenotype‐appropriate indications, GDMT use was substantial: among patients with LVEF < 50%, 90.9% were receiving a β‐blocker, 60.6% an ACE inhibitor, ARB, or ARNI, and 66.7% each an MRA and an SGLT2 inhibitor. Where therapy was not used, this frequently reflected intolerance or severely reduced eGFR in a population with a high prevalence of CKD. Among the 39 patients who avoided hospitalization, median NYHA class from III to II and mean weight decreased by 2.6 kg (5.7 lbs) at 30 days, consistent with effective decongestion. However, because these outcomes were assessed only in patients who did not require hospitalization, and some received IV diuretics during urgent care visits, they likely overstate the benefit across the full cohort.

Although torsemide and bumetanide retain high oral bioavailability, diuretic resistance in HF is driven largely by route‐independent mechanisms, including an elevated natriuretic threshold, reduced tubular drug delivery from impaired renal perfusion and neurohormonal activation, and distal nephron adaptation [12, 13, 14]. SC furosemide circumvents these limitations through predictable systemic absorption and sustained delivery that maintains drug concentrations above the natriuretic threshold. In pharmacokinetic studies, the pH‐neutral formulation achieved approximately 99.6% bioavailability relative to IV furosemide, with comparable 8 h urine output [8], supporting its use as an alternative to IV diuretics in hemodynamically stable patients. Our findings are broadly consistent with prior studies on SC furosemide. The Avoiding Treatment in Hospital With Subcutaneous Furosemide for Worsening Heart Failure (AT HOME‐HF) pilot trial, SC furosemide did not significantly improve the hierarchical primary endpoint of cardiovascular death, HF events, and change in NT‐proBNP at 30 days compared with usual care, although exploratory outcomes, including [weight loss, dyspnea, NYHA functional class], favored SC furosemide [15]. A 2024 systematic review of 20 studies including 687 patients found diuretic efficacy and natriuresis comparable to IV furosemide with a favorable safety profile, and reported potential reductions in healthcare costs and ED utilization [16], although many included studies used conventional furosemide administered subcutaneously rather than the pH‐neutral formulation.

SC furosemide may help address a gap in outpatient HF care between escalating oral diuretics and hospital admission. Outpatient IV diuretic programs offer one alternative, but they are available at approximately 18.7% of US hospitals, require infusion facilities, nursing staff, and patient travel, and have shown modest reductions in 30‐day readmission [17]. SC furosemide provides diuresis comparable to IV furosemide and can be self‐administered at home, which may extend outpatient decongestion to patients without access to such programs. In the Furoscix Real‐World Evaluation for Decreasing Hospital Admissions in Heart Failure (FREEDOM‐HF) case‐control study, patients presenting to the ED with worsening congestion who received SC furosemide instead of being admitted had 30‐day costs $16 995 lower per patient than matched cohorts, with no increase in subsequent HF‐related ED visits [18]. Although that study involved an ED population rather than a clinic‐based cohort like ours, its findings suggest potential economic benefits of this approach.

### 4.1 Limitations

This study is limited by its single‐center, retrospective design and small sample size, which reduces statistical power and generalizability. The absence of a concurrent comparator group limits causal inference; the within‐patient pre–post comparison of HFH is susceptible to regression to the mean, as SC furosemide was frequently initiated following a period of clinical deterioration. Selection bias may have influenced which patients were prescribed SC furosemide, and the retrospective design did not permit reliable ascertainment of the number of doses administered; exposure was defined by prescription, and patients confirmed administration on subsequent visits. Oral loop diuretic doses were concurrently increased at the SC furosemide initiation visit in 30.6% of patients, and GDMT was modified in 12.2%, which may have contributed to the observed improvements in NYHA class and body weight. Events at non‐participating institutions or those not reported in the EMR may not have been captured. Objective measures of congestion, such as KCCQ scores and natriuresis, were not available. Restricting the reporting of NYHA class and body weight changes to non‐hospitalized patients may introduce survivor bias and overestimate the observed improvements, although individual NYHA trajectories for all patients are shown in Figure 3. Despite these limitations, this study provides meaningful real‐world data on the safety and feasibility of SC furosemide in an ambulatory HF population and contributes to the growing evidence base informing its clinical use.

## 5 Conclusion

In ambulatory patients with HF who remained volume‐overloaded and symptomatic despite oral loop diuretic therapy, SC furosemide was well tolerated and associated with improvements in weight and functional status among patients who avoided hospitalization. Prospective controlled studies are needed to confirm its effects on hospitalization and to identify the patients most likely to benefit.

## Author Contributions

**Omar Jafar:** data curation, formal analysis, investigation, writing–original Draft. **Ann Harper:** data curation, formal analysis, writing–review and editing. **Angela Toepp:** project administration, writing–review and editing. **Amin Yehya:** conceptualization, methodology, supervision, writing review and editing.

## Funding

The authors have nothing to report.

## Ethical Statement

The study was approved by the local institutional review board, which waived the requirement for informed consent given the retrospective design.

## Conflicts of Interest

Dr. Amin Yehya receives consulting/speaking honoraria from Merck, AstraZeneca, Novo Nordisk, Bayer, Bridgebio, Mannkind, Kestra, Nuwellis, Kiniksa, and CVRx. The other authors declare no conflicts of interest.

## Data Availability

The data that support the findings of this study are available from the corresponding author upon reasonable request.
